# D-LIM: A neural network for interpretable gene–gene interactions

**DOI:** 10.1371/journal.pcbi.1014107

**Published:** 2026-03-23

**Authors:** Shuhui Wang, Alexandre Allauzen, Philippe Nghe, Vaitea Opuu

**Affiliations:** 1 Laboratoire de Biophysique et Evolution, UMR CNRS-ESPCI 8231 Chimie Biologie Innovation, PSL University, Paris, France; 2 LAMSADE, Universite Paris-Dauphine, PSL University, Paris, France; 3 Laboratoire de Biologie Structurale de la Cellule (BIOC), CNRS, Ecole Polytechnique, Institut Polytechnique de Paris, Palaiseau, France; Zhejiang University, CHINA

## Abstract

Recent advances in gene editing can produce large genotype–fitness maps for targeted genes, yet predicting the effects of mutations between genes remains challenging. Indeed, biochemical models require knowledge of underlying parameters and interactions, whereas machine learning methods typically lack interpretability, as they do not link model parameters to biological quantities. We introduce D-LIM, a neural network that infers low-dimensional fitness landscapes directly from mutation–fitness data. The distinctive feature of D-LIM is that it assumes genes act through independent gene-specific molecular phenotypes whose nonlinear interactions determine fitness. When this assumption holds, the model yields accurate predictions and interpretable effective phenotypes. Conversely, failure reveals that a low-dimensional model is insufficient. Applied to deep mutational scanning of metabolic pathways, protein–protein interactions, and yeast environmental adaptation, D-LIM achieves state-of-the-art predictive accuracy. The inferred phenotype–fitness landscapes reveal whether epistatic interactions can be captured by a low-dimensional continuous model and identify potential trade-offs. Moreover, D-LIM estimates mutational effects on the effective phenotypes, enabling weak extrapolation beyond the training domain. D-LIM demonstrates how simple structure constraints in a neural network can help inference and hypothesis generation in biology.

## Author summary

Understanding how organisms respond to genetic variation is essential for elucidating evolutionary principles. Advances in high-throughput sequencing now allow fitness measurements across thousands of genetic variants at once. These massive datasets are used to build models that explain mutational effects on fitness and predict outcomes of novel variants. A central goal of modeling is to extract biochemical insights and generate new hypotheses about the genotype-to-fitness relationship. However, modeling genotype-to-fitness relationships remains challenging due to the nonlinear, high-dimensional, and context-dependent nature of genetic effects on fitness. Ideally, one would use detailed biological knowledge to formulate hypotheses. However, they are often unavailable. In such a case, machine learning models may predict outcomes, but their opacity limits hypothesis generation. Here, we propose a neural network-based model that bridges this gap, based on a specific hypothesis: genes contribute independently to phenotypes, which are then combined through a function that determines fitness. Unlike conventional models, fitting data under this architecture directly evaluates the hypothesis. Moreover, the constrained architecture of the model yields interpretable phenotype predictions, enabling insights into genetic trade-offs and the global shape of the genotype-to-fitness map. This opens the possibility of uncovering modularity, redundancy, or epistasis patterns that shape fitness landscapes.

## Background

Understanding genotype-to-fitness maps is central to evolutionary adaptation and biomedical research in genetic diseases, the spread of infectious diseases, and drug resistance [1,2]. Genomic mutations affect certain molecular parameters, e.g., the stability of proteins, binding affinities, kinetic rates, expression levels. We refer to the latter as phenotypes. In turn, these parameters couple via metabolic and regulatory networks, ultimately determining the fitness of organisms. Fitness refers in this work to the growth rate (unless specified otherwise), as the latter is often used as a proxy for the degree of adaptation of one organism to its environment. Generally, multiple phenotypic parameters jointly contribute to fitness; the effect of a single mutation depends on the presence of other mutations. This phenomenon is called epistasis and causes mutation-to-fitness landscapes to be highly non-linear. Interactions between mutations in a gene are typically rugged and highly dimensional [3], due to the cooperative nature of residue interactions within proteins or RNAs. Likewise, mutations involved in physically interacting partners are typically rugged [4]. In contrast, combinations of mutations between different genes participating in a metabolic or regulatory network can be governed by smooth low-dimensional landscapes, as they reflect compositions of sigmoidal dependencies such as catalysis (Michaelis-Menten kinetics) or concentration binding curves [5]. While it is possible to build biochemical models of genetic interactions in well-characterized systems [6–8], this is rarely the case, because qualitative interactions or parameter values are unknown.

Experimental studies of genotype-to-fitness maps are done using large-scale screens called deep mutational scans [9–11], which consist of mutating genes and measuring the resulting fitness. With the progress of DNA-editing technologies, experiments can yield millions of fitness measurements as a function of mutations [12,13]. These recent progresses, notably based on CRISPR technologies, now allow the generation of libraries targeting several genes [14], where combinations of mutations can be tracked by next generation sequencing during selection experiments [15]. Notably, datasets containing mutations and corresponding fitness measurements for gene pairs and higher-order combinations are increasingly available [6,14,16–18], motivating the present study.

From such datasets, genotype–fitness relationships are currently inferred using two distinct approaches. The first type of approach is to devise a biochemical or biophysical model that defines fitness deterministically as a function of measurable quantities such as binding affinity or catalytic activity. For instance, in the study by Kemble *et al.* [6], the authors performed a mutational scan of two genes interacting via a metabolic pathway. They derived a model based on Michaelis-Menten kinetics and the structure of the pathway. The enzymatic activities of both genes determine fitness. Because the choice of model family dictates the role of each parameter, and these parameters are directly associated with a gene, this model is interpretable. However, identifying the correct parametric form and estimating its parameters are computationally demanding—Kemble et al. required 10^8^ Monte Carlo steps [19] for parameter optimization. Consequently, this approach remains difficult to extend to broader experiments, especially those involving complex or poorly characterized gene networks.

In contrast, machine learning (ML) models infer the mapping statistically, learning nonlinear dependencies directly from genotype–fitness data without assuming any specific form. Neural networks (NNs) capitalize on the increasing volume of data, resulting in a significant enhancement in prediction accuracy, particularly in the context of experimental mutational scanning of proteins [9]. Indeed, NNs are universal approximators [20], meaning that, instead of manually selecting a restricted parametric family, the learning process autonomously identifies complex relationships between genotype and fitness using high-dimensional internal representations. Furthermore, parameter optimization is highly efficient via the backpropagation algorithm. However, interpreting how the representation influences the predictions and its relationship with phenotypes is unclear. In ML, linear regression models are considered to be the most interpretable, but they offer limited predictive power [21], especially in the context of interactions between mutations, which are non-linear. Together, biochemical and ML models represent opposing ends of the modeling spectrum, motivating the search for methods that are both interpretable and data-driven.

Interpretability is a current challenge in ML in general, in biology in particular. In the context of deep mutational scan experiments, the LANTERN approach constrains the latent representation of mutations to be additive, thereby facilitating the interpretation of the model parameters [22]. Two mutations with latent representation vectors pointing in the same direction should have similar effects, while the magnitude of these vectors informs on the strength of the mutation. Nevertheless, the specific role of the dimensions composing the coordinates remains unclear regarding predictions or phenotypes. From a biophysical perspective, Tareen *et al.* introduced MAVE-NN, which simplifies the development of mechanistic models for genotype-to-phenotype maps [23]. In MAVE-NN, users specify the type of map—ranging from additive to NN-based—between the genotype to fitness with a latent phenotype intermediate. As a result, while the latent phenotype is never directly measured, it can be interpreted as an effective phenotype. This approach presupposes that a single effective phenotype summarizes the influence of all genotypes. The approach was further extended for interpretability, by locally using models with a functional form akin to biochemical models [24]. Faure and Lehner also developed this line of reasoning by employing multiple latent phenotypes [25]. In a different approach, GenNet introduced an interpretable NN designed to study systems of genes or metabolic pathways [26]. Its architecture is informed by *a priori* biological knowledge, with neuron connectivity defined by gene annotations, pathways, cellular expression, and tissue expression. However, this method necessitates rich information about the system.

Overall, a central aspect of interpretability in genotype–fitness models is to identify certain variables of the models to biophysical or phenotypic quantities, or more generally to be able to infer some information about these quantities without measuring them directly. In most ML approaches, mutations are represented as high-dimensional vectors capturing complex genetic dependencies, but the biological meaning of each dimension is unclear.

To fill this gap, we introduce D-LIM (Direct-Latent Interpretable Model), a model for gene-gene interactions that learns from genotype-to-fitness data. It is built on the assumption that each mutation independently determines an effective phenotype, and that fitness arises from a non-linear but smooth combination of these effective phenotypes. The rationale behind this 2-stage model is that many biological processes can be modeled by smooth continuous functions of certain parameters (binding constants, production rates, expression levels). The problem is that such a relationship cannot be directly detected in mutation-to-fitness maps because: (i) the relationship between mutations and intermediate (unobserved) variables can be highly rugged, and (ii) it is not obvious to deconvolve the effect of combined mutations acting on different parameters. In particular, it was shown that even single-peaked Gaussian phenotype-fitness landscapes can lead to rugged mutation-to-fitness landscapes when approaching a fitness extremum [27]. The purpose of D-LIM is to detect whether such a relationship exists, and if so, to introduce intermediate gene-associated variables, called effective phenotypes, associated with genes, to determine how mutations affect the effective phenotypes, and automatically find the smooth relationship between effective phenotypes and fitness.

We show that D-LIM, due to its architecture, offers interpretable features. We apply it to three mutational scanning experiment datasets, where it reaches state-of-the-art accuracy in fitness prediction. In the case of gene networks, the model indeed reproduces epistatic interactions from the non-linearity of smooth phenotype–to-fitness landscapes, whereas it fails at explaining epistasis between physically interacting proteins beyond the linear regime, confirming the high-dimensional character of molecular interactions. When successful, D-LIM ranks mutations to effective phenotypes even for non-monotonous relationships, identifies phenotypic trade-offs, and extrapolates the fitness prediction outside from the training data domain.

## Datasets

### Synthetic datasets

To evaluate model performance under controlled conditions, we generated synthetic datasets from three well-established biophysical and evolutionary models:

- The mechanistic model proposed in [6] describes a two-enzyme linear metabolic pathway in which the growth rate (fitness) *F*(*X*,*Y*) depends on the activities of two genes, araA and araB. We refer to this model as the linear regulation model. *X* and *Y* represent the enzyme activity of mutants of araA and araB, respectively, and *F*(*X*,*Y*) quantifies the resulting flux-dependent growth rate.
- The tilted Gaussian (Fisher’s Geometric) model represents a simplified evolutionary landscape where each genotype maps to a single phenotype [28]. Restricted to two genes, the fitness function *F*(*X*,*Y*) is modeled as a multi-variate Gaussian centered around an arbitrarily chosen optimal phenotype, tilted to introduce directional selection. *X* and *Y* denote two independent phenotypic axes contributing to the overall fitness.
- The gene regulatory cascade model captures the epistatic interaction between two transcription factors, LacI and TetR, in an *E. coli* signaling cascade [7]. The fitness function *F*(*X*,*Y*) reflects how combinations of transcriptional activities *X* for LacI and *Y* for TetR affect downstream gene expression and, consequently, cellular fitness.

For each model, we simulated two genes *X* and *Y*, each carrying 30 distinct mutations with phenotypic values randomly sampled from the interval [0,5]. All pairwise combinations of mutations (30×30=900) were generated to form the complete mutational landscape. Corresponding fitness values were then computed using the respective biochemical or evolutionary model-specific formulations (see details in S1 file for explicit equations).

### Two-gene metabolic pathway dataset

This dataset involves two genes in the metabolic pathway of L-arabinose utilization: L-arabinose isomerase (AraA) and L-ribulokinase (AraB) (See Fig 4A). These enzymes convert L-arabinose into L-ribulose-5-phosphate, which is critical for cellular growth. Mutations were systematically introduced in the promoter regions of both AraA and AraB genes (including 12 positions for each), enabling a comprehensive analysis of individual and combined genetic perturbations, covering a total of 111 mutations per gene promoter. The measurements were performed in three distinct environments (Env1, Env2, Env3) that differ by the concentration of inducers promoting the synthesis of both enzymes. Env1 had inducers for both genes, while Env2 and Env3 had respectively 5 ng and 200 ng of the araA inducer but without the araB inducer. The data consists of 1,369 mutation combinations and the resulting fitness (measured as growth). The phenotypes are the activities of the enzymes AraA and AraB, which are not measured.

### Two physically interacting proteins

In [16], Diss and Lehner quantified the effect of mutations on a system of two proteins, called FOS and JUN that interact via their leucine zipper, a 32-residue helix (Fig 7A). Consequently, the fitness measured is defined based on identified phenotypes, specifically the physical binding between the two proteins. To investigate genetic interactions, two mutations were introduced for each pair of mutants—either one mutation in each gene expressing the proteins or both mutations in one gene. The fitness for each pair of mutations depends on the binding strength of the mutated proteins quantified by the protein-protein interaction (PPI) score, resulting in over 120,000 data points. This dataset delves into two types of interactions: the first is fitness, which is mainly driven by the concentration of protein complexes, while the second is epistasis, resulting from specific structural interactions between the two protein helices.

### Yeast strains adaptation across multiple environments

Kinsler *et al.* [17] measured the fitness of 292 yeast strains across 45 different environments by varying factors such as the concentrations of NaCl, KCl, glucose, fluconazole, and geldanamycin in the growth medium, as well as the transfer time during the experiments. For each environment, the fitness of all mutants was determined by monitoring their populations throughout the adaptation experiment using barcode sequencing. In total, 13,140 mutation-environment fitness measurements were obtained. Two types of environments were identified: those yielding small fitness perturbations (7,300 data points), that are referred to as subtle; and those yielding strong fitness perturbations (5,840 data points).

## Results

### D-LIM

**Fig 1:**
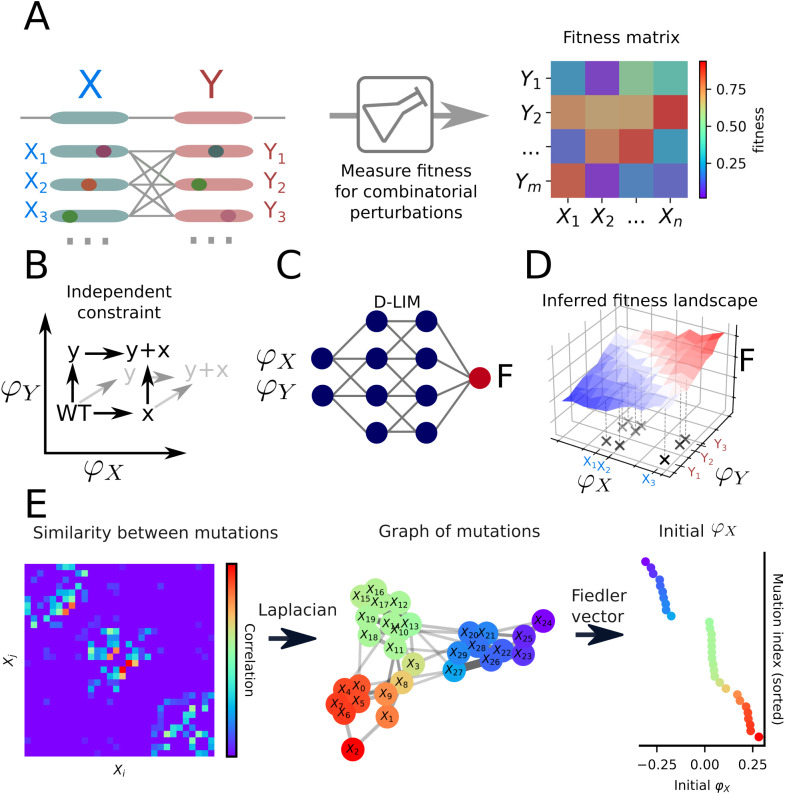
Overview of the D-LIM framework. **A)** Data acquisition for a pair of genes *X* and *Y* where fitness is measured across a combination of mutations. The fitness data is structured as a matrix in which each pair of rows represents the two mutations of two genes in the same genetic background, and each pair of columns follows the same representation. **B)** Phenotype independence. Each dimension represents an independent gene effective phenotype φ. Consequently, mutations in such a constrained space are independent (represented by the dark arrows). If mutations did not affect phenotypes independently, they would vary along all dimensions at once (as represented by the grey arrows). **C)** D-LIM architecture. D-LIM takes one phenotypic value per gene variant and processes it through a feed-forward neural network to predict the fitness. **D)** The constrained resulting fitness landscape. The fitness is mapped to the constrained phenotype space where combinations of gene variants are organized in a grid. **E)** Spectral initialization of the effective phenotypes φ. To initialize the phenotype φ, we first compute Pearson correlations between mutation fitness profiles from panel A to quantify similarity between mutations. We then construct the Laplacian from this similarity matrix to encode these relationships in a graph structure. Its Fiedler vector (the eigenvector associated with the second smallest eigenvalue) then provides a one-dimensional embedding that serves as the initial. These values capture in a one-dimensional space the topology of the graph, as represented in the right scatter plot, where mutations are sorted by φ. The same procedure is applied for *X* and *Y*.

D-LIM is a neural network (NN) designed to learn genotype-to-fitness maps from a list of genetic mutations and their associated fitness when distinct biological entities are referred to here as ‘genes’ for simplicity (Fig 1A). We describe in the next section its architecture, while in the following section, we demonstrate in simulated datasets that D-LIM enables us to recover phenotypic values. Finally, we show that our model generalizes beyond already explored mutations, akin to mechanistic models. To facilitate practical use, we provide a Python package available on PyPI, designed for easy installation, data formatting, and generation of interpretable outputs. The link is in the Availability of data and material section.

#### D-LIM architecture.

The model comprises three levels of description: mutations (genotypes), phenotypes, and fitness. Phenotypes are understood here in a very general sense as fitness-determining parameters, which may represent low-level molecular properties (e.g., binding), and higher-level measurable properties (e.g., metabolic flux). The space spanned by the phenotypic values is termed the phenotype space. We connect these levels of description via a genotype-to-phenotype map and a phenotype-to-fitness map. The genotype-to-phenotype map encodes the hypothesis that mutations participating in different genes do not interact when determining phenotypes, though mutations within the same gene can interact (Fig 1B). In the NN, this corresponds to associating each mutation with a single phenotypic value, while the phenotype-to-fitness map is learned using an NN (Fig 1C), leading to a relationship of the form $\hat{F}=f(\varphi_{1},\varphi_{2},...)$ (Fig 1D). The training of D-LIM consists of two steps: (1) initializing the genotype-to-phenotype map by assigning initial values to $\varphi_{i}$, and (2) simultaneously training all parameters, including the $\varphi_{i}$.

The initialization starts with the assumption that mutations with the same phenotypic value yield similar fitness values in a matching genetic background (in combination with the same mutations). We called this procedure spectral initialization, which for one gene follows these steps (see Fig 1E): (i) Similarity calculation: compute similarities between mutations using fitness values from the training data; (ii) Spectral decomposition: compute the eigenvector corresponding to the second smallest non-zero eigenvalue of the Laplacian of the similarity matrix, which is called the Fiedler vector. The efficiency of this approach depends on the structure of the data. To measure how well the data is structured, we use the spectral gap, that is the difference between the smallest and the second smallest eigenvalues. (iii) Initialization of φ: If the spectral gap < 0.95 and spectral gap > 0.05, the values in the Fiedler vector are used as the initial phenotype value for each mutant. Otherwise, we use the random Xavier-Glorot method [29], see section for more details.

Once the genotype-to-phenotype is initialized, we simultaneously train the NN and refine the $\varphi_{i}$ parameters using the Adam optimizer [30] in the Pytorch framework [31]. The parameters are updated using the Negative Gaussian Log-likelihood loss function which accounts for the measurement noise [30]. To favor a smooth landscape, we applied *L*_2_ regularization to the φ [32]. During training, D-LIM refines the phenotypic values while simultaneously learning to predict fitness (Fig A in S1 File).

Due to the gene-to-phenotype constraints, D-LIM is more rigid than existing ML approaches. For instance, in LANTERN, mutations are represented in an unconstrained latent space, where mutations may span multiple dimensions, but without being linked to any specific biological entity.

#### Phenotype inference.

In our genotype-to-phenotype map, genes are assumed to act independently in determining the phenotypic value. While our phenotypes φ are not measured but inferred, they are thought to be proxies for measurable biological phenotypes. We now show on simulated data that using our hypothesis infers phenotypes related to the true underlying phenotypes.

To perform our test, we employed three synthetic datasets (details in Section): (i) the two-enzyme metabolic pathway [6]; (ii) a tilted Gaussian model [28]; (iii) a gene regulatory cascade model [7]. Then, for each dataset, we trained D-LIM with half of the samples. To demonstrate the importance of the spectral initialization, we trained a second model where the φ were randomly initialized.

**Fig 2:**
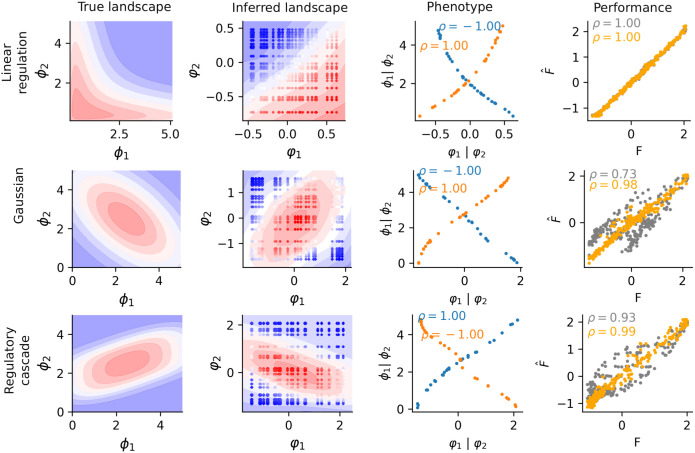
Simulated landscapes. From left to right, the plots display the theoretical fitness landscapes in a blue-to-red color gradient (first row: linear regulation of two genes, second row: geometric model with Gaussian expression, and third row: regulatory cascade model); the fitted landscape using spectral initialization; a scatter plot of true versus inferred phenotypes. Note that effective phenotypic values are inferred up to a global scaling factor (positive or negative, as in principal components analysis), which can in particular result in a reversed x-axis as seen here; a scatter plot of predicted versus true fitness values, with spectral initialization (orange) and without (grey).

For the metabolic model by Kemble *et al.* [6], we observed a good agreement with the fitness (Pearson correlation ρ=1.00, *R*^2^ = 0.91). While the fitness landscape spanned by the trained phenotype space is different from the original one (Fig 2), the inferred phenotypes and the corresponding measured phenotype (enzyme activity) were strongly correlated (Spearman correlation reached ρ≈1). We observed that both initialization strategies led to a monotonous relationship between true and inferred phenotypes and similarly high predictive power.

For the Gaussian and the regulatory cascade model, we first tested the random initialization for the φ, which led to poor phenotype inference with rough landscapes (Fig 2). We determined that the asymmetry of the shape is the cause of the loss of accuracy. Indeed, the shape of these models induces a strong dependency on the coordinates between the inferred phenotype axes φ and the fitness *F*. For example, in the non-rotated zero-centered Gaussian model, *e.g.,* F(x,x)=F(x,−x), whereas the rotated Gaussian does not allow for such symmetry. Therefore, the genotype-to-phenotype map has more optimal solutions for the non-rotated symmetrical Gaussian model (similarly for the cascade model), which may cause the loss of accuracy of D-LIM fitting.

Applying the spectral initialization showed a clear improvement in prediction accuracy and in inferred phenotypic values for the non-monotonous landscapes. With the tilted-Gaussian model, the prediction accuracy increased from ρ=0.73 to ρ=0.98, respectively *R*^2^ = 0.68 to *R*^2^ = 0.98 (see Fig 2). With the cascade reaction, we observed consistent improvements, where the accuracy increased from ρ=0.93 to ρ=0.99, respectively *R*^2^ = 0.80 to *R*^2^ = 0.99. For the true phenotypic reproduction, we showed that the spectral initialization overcame the limitation of non-monotonous underlying landscapes ϕ (Spearman correlation |ρ|=1 for both models, see Fig B in S1 File), allowing us to recover the shape of the underlying true landscape.

We have shown that our approach enables us to recover a monotonous relationship between the inferred and true phenotype, which is non-obvious given that data only provides genotype-to-fitness relationships.

#### Phenotypic measurement process for fitness extrapolation.

Extrapolation of the prediction outside of the data domain is typically not expected for NN models. In contrast, mechanistic models can be fitted within a certain data domain, so that in turn, the model parameters can be used for prediction outside of the initial domain. Given that D-LIM retains some of the constraints of mechanistic approaches, we surmised that the learned biochemical parameters may be used for extrapolation within a certain range beyond the training data. The extrapolation strategy relies on the relationship between inferred phenotypes and actual phenotypes, requiring the measurement of actual phenotypes for a subset of individual genes. Indeed, when experimental measurements of the phenotypes are available for some of the mutants, the inverse relationship $\omega^{-1}(\phi )=\varphi$ between inferred and actual phenotypes can be fitted by a smooth function. Fitness predictions can then be extended to new mutants by measuring their actual phenotypic values φ associated with single mutations, then deducing their latent phenotypic value φ via $\omega^{-1}$.

**Fig 3:**
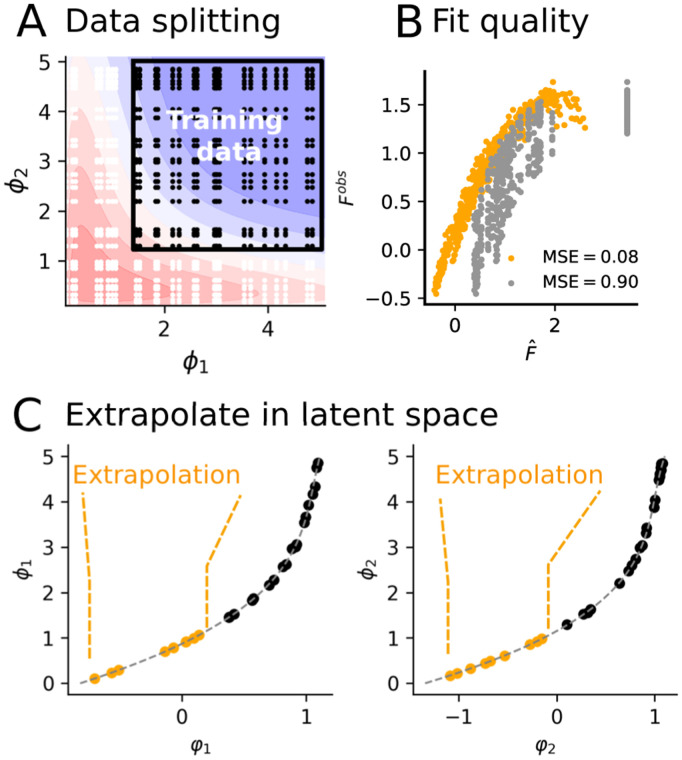
Extrapolation with unseen mutations and their measured phenotypes. **A)** Fitness landscape from the mechanistic model of [6]. Black points indicate training data and white points indicate validation data. **B)** Fit quality. Conversion of true phenotypic values into inferred phenotypes by D-LIM for extrapolation. Yellow points represent cases without additional true phenotype values, while grey points use them. **C)** Extrapolation using phenotypic measurements: $\phi_{1}$ (left) and $\phi_{2}$ (right). Black points ($\phi_{1}$, $\varphi_{1}$) are used to parameterize the polynomial fit in a dotted grey line. Orange points are the converted $\phi_{1}$ and $\phi_{2}$ only seen in the validation, which are converted into inferred phenotypes using the polynomial fit.

To illustrate this approach, we simulated an experiment with the linear regulation model [6], where fitness is controlled by two phenotypes X and Y—enzyme activities by AraA and AraB. We restricted the training data to pairs of mutations yielding low fitness (black square in Fig 3A), whereas mutation combinations outside of this domain were left for validation. For the sake of comparison, we first tested a naive extrapolation strategy, directly using the model to predict fitness for combinations involving never-observed mutations. The prediction displayed poor accuracy (ρ=0.63, *R*^2^ = 0.16) (Fig 3B). Then, we applied our strategy using a third-order polynomial to fit the relationship between $\varphi_{1},\varphi_{2}$, and their respective phenotypic values $\phi_{1},\phi_{2}$, as shown in Fig 3C. Using the resulting phenotypes as an input for the NN yielded high fitness prediction accuracy (MSE = 0.08, ρ=0.98, *R*^2^ = 0.85), as shown in Fig 3B. This extrapolation technique also worked well for non-monotonous fitness functions (see Fig C in S1 File).

Overall, the independence assumption allows D-LIM to extrapolate fitness predictions beyond the training data using phenotypic measurements, which would not have been straightforward to implement with other ML approaches. Although this extrapolation strategy requires additional measurements, it could be of practical interest because it relies on single mutation data instead of combinations of mutations.

### Predictive and interpretable constrained architecture

We now apply D-LIM to interpret phenotypes and reveal potential trade-offs across three mutational scanning datasets: a two-gene metabolic pathway, a system of two physically interacting proteins, and yeast strains adaptation across multiple environments.

#### Revealing hidden phenotypes from gene-to-fitness.

We used D-LIM to predict fitness of Kemble *et al.* dataset, using two phenotypes $\varphi_{A}$ and $\varphi_{B}$, corresponding to enzymes AraA and AraB, respectively. We trained the model on 70% of the data and evaluated its accuracy on the remaining 30%—we repeated 10 times across random data splits to obtain the average correlation. In Env1 and Env2, D-LIM predictions showed strong agreement with measured fitness (ρ=0.99, *R*^2^ = 0.92 and ρ=0.98, *R*^2^ = 0.92, respectively with p-value $\leq 10^{-5}$). Next, we tested the predictive accuracy on epistasis, which is the deviation in the fitness of a pair of mutations from the sum of their individual fitness E(a,b)=F(a,b)−F(a)−F(b). D-LIM predicted similarly well epistasis with ρ=0.97 (p-value ≤0.005, *R*^2^ = 0.90).

**Fig 4:**
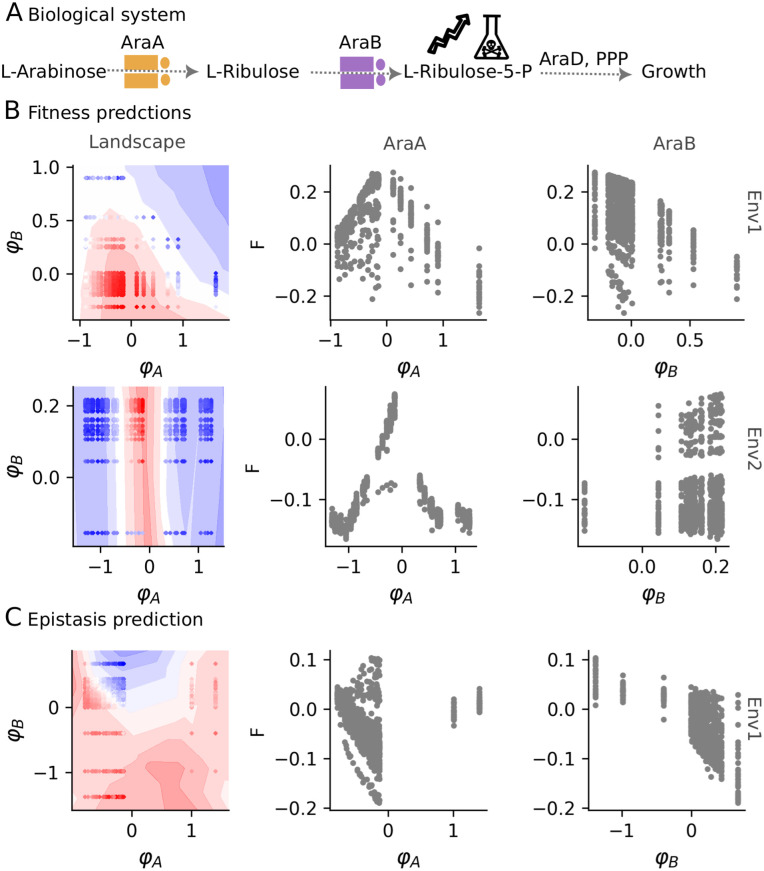
Gene-gene interaction pathway test case. **A)** L-Arabinose pathway of *E. coli.* The figure is adapted from *Kemble et al.* [13]. **B)** Fitness prediction in two environments. From left to right, we show the predicted fitness landscape represented by a blue-to-red color gradient contour for the prediction, where $\varphi_{A}$ and $\varphi_{B}$ are the inferred phenotypes for mutations in genes AraA and AraB. The points indicate the pairs of mutations colored by the experimentally measured fitness. Next, we display the relationship between the measured fitness and the two inferred phenotypes. The first row corresponds to analyses in Env1, while the second row shows Env2. **C)** Epistasis prediction. This row illustrates the accuracy, landscape, and correlation between inferred phenotypes and epistasis in Env1.

In the inferred landscapes, we observed qualitative differences which are quantified by analyzing the variation of fitness along the inferred phenotypes. In Env1, the fitness varies across both inferred phenotypes, indicating that both genes influence the measured fitness. However, D-LIM revealed a trade-off in AraA, that is varying $\varphi_{A}$ beyond a threshold reduces the fitness (Fig 4B), whereas fitness varies monotonously along $\varphi_{B}$ (|ρ|=0.43 with p-value $<10^{-5}$). For epistasis, it varies monotonously only along $\varphi_{B}$ (|ρ|=0.40 with p-value $<10^{-5}$) (Fig 4C). In contrast, in Env2, the fitness varied along $\varphi_{A}$ with a similar trade-off; but not along $\varphi_{B}$ (|ρ|=0.08, p-value = 0.003). This means that AraB activity is anticipated to not vary between mutants. Indeed, since no AraB inducer was used in Env2, we only expected basal effects. This conclusion was reached because of the independence of phenotypes assumption imposed in D-LIM, which allowed us to separate the contribution of each phenotype.

**Fig 5:**
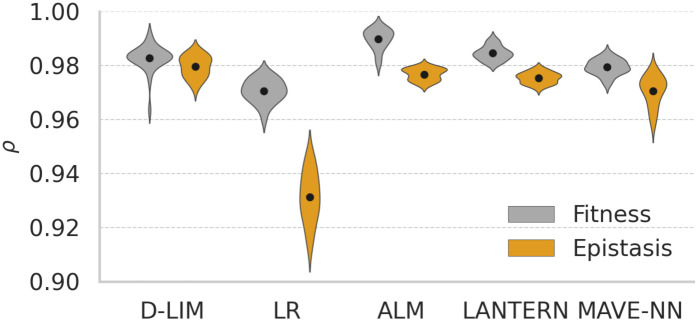
Comparing prediction across models. Violin plot of Pearson correlations for random cross-validation across 30 runs. 70% of the original data was used as training dataset and the remaining 30% as the validation set. The black dot represents the average Pearson correlation for each model on the fitness or epistasis dataset.

Does imposing the constraint of independent phenotypes result in a trade-off with predictive accuracy? To explore this, we compared D-LIM to three analogous models that do not impose constraints on their latent spaces: a) the linear regression (LR), b) LANTERN, which is based on the Gaussian process, c) the Additive Latent Model (ALM) using an NN, and d) MAVE-NN that is biophysics-based (see section for details). In Kemble *et al.* dataset, the fitness is largely additive (see Fig D in S1 File), which can be seen from the performance of the LR (on average ρ=0.97) and ALM (additive latent model, ρ=0.99); so the challenge is to predict epistasis. As expected, all other models performed significantly better than LR for epistasis prediction (Fig 5). For MAVE-NN, we choose to illustrate the additive genotype-to-phenotype map offered, which essentially corresponds to a regression. For the NN-based models, we did not observe any striking loss of fitting performance when comparing D-LIM with its version with the unconstrained latent space (called ALM here).

**Fig 6:**
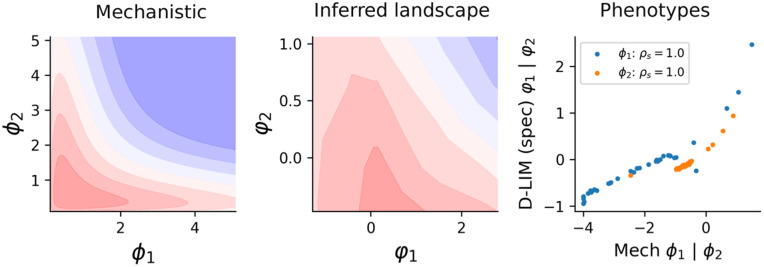
Capturing mechanistic hypotheses. (left) The mechanistic model obtained from [6] is displayed in contour. (middle) The learned landscape of D-LIM trained on all the data points of the Env1. (right) The D-LIM inferred phenotypes are compared with the phenotypes inferred earlier from the mechanistic model (extracted from [6]).

Finally, we showed that the inferred phenotypes are consistent with the biophysical hypotheses established earlier for this system, as implemented in the mechanistic model in [6], see details in S1 file. To validate this, we compared the inferred φ here with the ones inferred earlier for the mechanistic model for the Env1 since the other two are depleted with AraB inducers. Fig 6 shows that the inferred phenotypes are consistent with the ones inferred with the mechanistic model, where Spearman’s correlations $\rho_{s}=1$ for both phenotypes. This demonstrates that D-LIM is capable of revealing mechanistic insights, which were explicitly modeled in [6].

Our results show high predictive performance for both fitness and epistasis, suggesting that the hypothesis of phenotype independence is largely satisfied in this system. Interpretation of the inferred landscapes recovered mechanistic insights, confirming a trade-off in one enzyme that produces L-ribulose-5-phosphate, its accumulation leads to toxicity, resulting in inhibiting the growth of the cell [6].

#### Interpreting mutations from physical interactions.

In this section we propose a negative control system that involves highly dependent phenotypes, which contradicts our foundational hypothesis. For this, we considered the JUN-FOS protein complex where fitness perturbation upon mutations on both proteins has been measured [16]. Thus, we used D-LIM to search for a smooth landscape that involves two independent effective phenotypes ($\varphi_{FOS}$, $\varphi_{JUN}$) which capture the observed fitness and epistasis variations.

**Fig 7:**
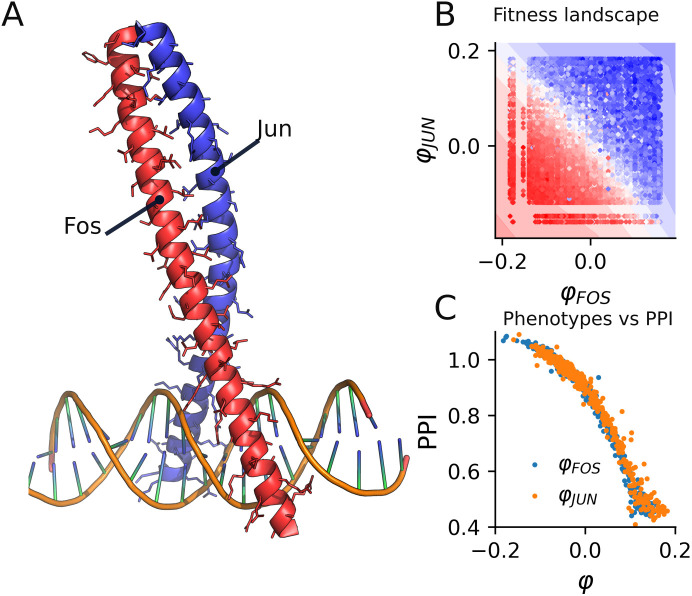
Predicting protein-protein interactions. **A)** JUN and FOS proteins physical interaction modulated by amino acids at the interface, with a zoom on the interacting part. **B)** The fitted landscape (contour) modeling the variation of fitness where dots are pairs of mutations colored by the observed fitness value whereas the contour coloring is the prediction. **C)** Experimental PPI scores for single mutants are represented against the inferred phenotypes from D-LIM.

We started with fitness. For this case, the spectral initialization yielded a spectral gap $\lambda_{2}>0.95$; therefore, we initialized the effective phenotypes randomly. Then, we equipped D-LIM with two layers of 64 neurons to predict the fitness *F*. D-LIM has then been trained on 70% of the data and tested against the remaining 30%. Our prediction quality matched that of the thermodynamic model proposed alongside the data, with D-LIM explaining 93% of the variance. Fig 7B displays an additive smooth inferred landscape where fitness varies across mutations on both proteins.

We then compared the inferred φ values with the single mutation PPI score, obtained experimentally, which quantifies the strength of the physical interaction (Fig 7C). D-LIM consistently recovered the sigmoidal relationship between the fitness and the physical interaction, with an average ρ=0.97 ($p\text{-value}<10^{-5}$, *R*^2^ = 0.93). We also compared the prediction accuracy on the fitness from state-of-the-art methods. D-LIM achieved comparable results as ALM, LANTERN, and MAVE-NN (*p-value* < 0.05, see Table 1).

**Table 1:** Comparison of performance between D-LIM and state-of-the-art methods on simulated and experimental datasets, results are mean ± standard deviation of Pearson correlations on 30 independent executions. NA (for Non-Attributed) means that the method doesn’t apply to the case.

		LR	ALM	LANTERN	MAVE-NN	D-LIM
Simulated dataset	Linear regulation	0.97 ± 0.00	0.99 ± 0.00	0.99 ± 0.02	NA	0.99 ± 0.00
	Gaussian	0.74 ± 0.00	0.99 ± 0.01	0.95 ± 0.03	NA	0.99 ± 0.00
	Regulatory Cascade	0.85 ± 0.00	0.99 ± 0.00	0.94 ± 0.04	NA	0.99 ± 0.00
Yeast dataset	Fitness Env1	0.97 ± 0.01	0.99 ± 0.01	0.98 ± 0.01	0.98 ± 0.01	0.98 ± 0.01
	Epistasis Env1	0.93 ± 0.01	0.98 ± 0.01	0.98 ± 0.00	0.97 ± 0.01	0.98± 0.01
Protein-protein interactions dataset	Fitness	0.93 ± 0.00	0.96 ± 0.00	0.96 ± 0.00	0.95 ± 0.00	0.97 ± 0.00
Mutation-environment dataset	Env subtle	0.92 ± 0.00	0.95 ± 0.01	0.96 ± 0.01	NA	0.94 ± 0.01
	Env strong	0.58 ± 0.02	0.86 ± 0.05	0.85 ± 0.06	NA	0.87 ± 0.03

Next, we tested D-LIM on epistasis. Following the same protocol as above, we fitted a D-LIM model on the epistatic values derived from the same data. However, D-LIM failed at capturing epistasis (ρ=0.22 with p-value $<10^{-5}$, $R^{2}=-17.21$, Fig E in S1 File). This result suggests that epistasis cannot be decomposed into independent effective phenotypic values, but requires higher-order models.

While the fitness is mostly additive, the remaining epistasis is difficult to predict. D-LIM did not find independent effective phenotypes capturing observed epistasis. This is the result of the cooperativity between these two proteins at the side-chains interaction level, which is completely omitted in D-LIM. While there is not much value for an already characterized interaction, in less characterized cases where a physical interaction is only putative, the presence of a smooth gene-associated landscape would point to functional interactions being mediated by other genes, whereas its absence would point to direct physical interactions.

#### Predicting fitness variation across environments.

**Fig 8:**
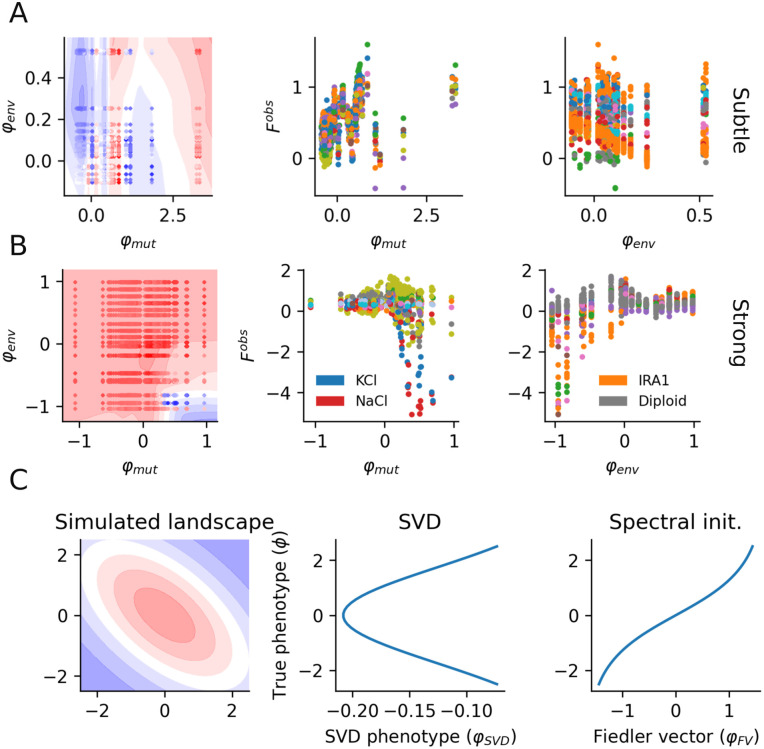
Predicting mutations across various environments. **A)** Subtle environments from [17]. From left to right, we show the inferred landscape, the inferred phenotype (corresponding to mutations) against observed fitness but colored by environments, and the inferred environment phenotype is colored by the corresponding mutations. **B)** The second row presents the same plots for the strong environment as defined in [17]. **C)** The SVD approach applied to the Fisher geometric model (left) is shown, where the first eigenvector (interpreted as inferred phenotypes) is plotted against the true phenotype. Finally, we compare this with the initial guess obtained from the spectral initialization procedure (Fiedler vector).

To apply D-LIM, we studied the yeast strains’ adaptation across multiple environments, modeling the yeast mutations with one phenotype $\varphi_{mut}$ and the environment with a second phenotype $\varphi_{env}$. We started by analyzing the 13,140 data points of the subtle environments (70% for training and 30% for validation). In D-LIM, we used two hidden layers of 64 neurons, and we initialized the φ using the spectral initialization procedure. Our results showed a strong correlation with the experiments, achieving ρ=0.93 (p-value $\leq 10^{-5}$, *R*^2^ = 0.78). The fitness landscape inferred from the analysis confirmed the mitigated effect of the environment on fitness (Fig 8A). In contrast, D-LIM suggests the presence of local optima in the mutations. However, we noticed that at the level of individual genes, no clear trade-off could be identified (Fig F in S1 File). The mutations involved in the high-fitness region are in the IRA1 and IRA2 genes, which are partly responsible for the adaptation to glucose-limited environments [33]. To demonstrate the performance of D-LIM, we compared results from D-LIM to ALM and LANTERN for the prediction of fitness in weak environments. All models showed similar performance to the baseline (see Table 1), because the landscape is highly additive.

For the strong perturbations, we observed a similar prediction accuracy (ρ=0.86 with p-value $\leq 10^{-5}$, *R*^2^ = 0.62), but in contrast with above, the environments now are responsible for the largest variation in fitness (Fig 8B). The results remain the same for the other state-of-the-art methods, the Pearson correlation for all models is around 0.86. Notably, the environments varied continuously across the phenotype $\varphi_{mut}$, in NaCL and KCl, meaning that the inferred phenotype is sensitive to salt concentration.

Kinsler *et al.* proposed an interpretable modeling technique based on the singular value decomposition (SVD) applied to the fitness dataset. Specifically, they constructed a matrix F, where the entries represent the measured fitness of various mutation-environment combinations. Applying the SVD to this matrix F allowed them to extract vectors representing the mutant phenotypes and vectors representing the environmental weights (respectively being the left and right eigenvectors of the SVD). They reproduce fitness using as few as eight phenotypes, corresponding to the eight most important left eigenvectors. Because 95% of fitness variability is explained by the first component, a linear relationship appears between the mean fitness and the inferred phenotypes (Fig G in S1 File). This approach however might not represent the structure of the landscape well if this landscape is not monotonous; therefore missing potential trade-offs. To demonstrate this, we used the Fisher geometric model to create an artificial dataset from which we inferred phenotypes $\varphi_{SVD}$ using the SVD, which we compared with the true phenotypes ϕ. Fig 8C shows that the relationship between $\varphi_{SVD}$ and ϕ is not monotonous. In contrast, using spectral initialization, the initial guess of the $\varphi_{FV}$ (the Fiedler vector) is already well related to true phenotypes, allowing us to provide a correct interpretation of the phenotypes.

To our knowledge, D-LIM is the only approach that offers a robust modeling of non-monotonous and non-symmetric fitness landscapes, enabling us to detect potential trade-offs. These trade-offs are generally missed by methods such as the SVD proposed earlier.

#### Robustness and stability analysis.

Interpretability requires accuracy of the fitness prediction and robustness of the phenotype inference. To demonstrate this, we first assessed the fitting performance of DLIM across datasets with and without spectral initialization. We then tested the robustness of the phenotype inference over multiple realizations of the learning process.

Beforehand, we verified that D-LIM with spectral initialization generally performs better than without it, and thus should be used by default for comparison with other models. We ran D-LIM 30 times and recorded the prediction-validation correlation with early stopping, with or without spectral initialization. Over 8 datasets (including simulated and real ones), spectral initialization consistently improved prediction accuracy (p-value < 0.05; Fig H in S1 File), except for the linear regulation system and the yeast epistasis dataset, for which it performed equally. Although spectral initialization may lead to better predictions even before training, we noted that this was not the case and that improvement was revealed only after training (Fig I in S1 File).

**Fig 9:**
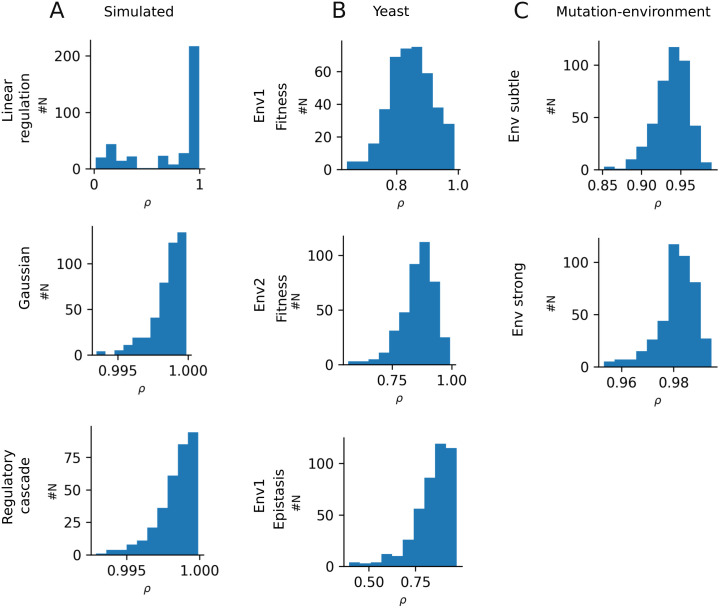
Robustness analysis on simulated datasets and experimental datasets. It displays the distribution of pairwise Pearson correlation between the inferred phenotype ($\varphi_{X},\varphi_{Y}$) across 30 independent model training. **A)** Simulated datasets: Linear regulation, Gaussian, and regulatory cascade system. **B)** Yeast datasets: fitness data in Environment 1 (Fitness Env1), fitness data in Environment 2 (Fitness Env2), epistasis data in Environment 1 (Epistasis Env1). **C)** Mutation-environment dataset: all mutation data in subtle environments (Env subtle) and all mutation data in strong environments (Env strong).

To demonstrate that inference is stable, we show here that the inferred phenotypes are robust across multiple runs, although the NN parameters are randomly initialized. We trained D-LIM 30 times on both the artificial and real datasets; then extracted $\varphi_{x}$, $\varphi_{y}$ from each model. We then computed pairwise Pearson correlations between all 30 $\varphi_{x}$ and between all 30 $\varphi_{y}$ (yielding two times 435 correlations), summarized in Fig 9. The high average correlation (ρ=0.87 for yeast data and ρ=0.95 for mutation-environment data) indicates that D-LIM converges to the same inferred phenotypes, which yields robust and reproducible landscape predictions.

In summary, the landscape inferred by D-LIM reaches the accuracy of existing machine learning approaches, but additionally infers genotype-phenotype relationships robustly, showing that the hypotheses imposed by D-LIM enable a further step in interpreting biological data compared to hypothesis-free models.

## Discussion

Genotype-to-fitness maps are central to understanding and predicting evolutionary adaptation. These maps can be fitted with biochemical models, which are expected to be predictive and interpretable, as the parameters of the model have a meaning such as binding constants or reaction rates. However, they require prior knowledge, which is rarely available, given the very large number of molecules and interactions present in cells. In contrast, ML models can be predictive without knowledge of the mechanisms, but at the cost of interpretability. In this work, we proposed D-LIM, a model that encodes biological hypotheses in the ML architecture: a relationship between mutations and genes, the constraint that one gene affects only one phenotype, and that fitness results from a continuous landscape as a function of phenotypes. D-LIM thus mimics the mathematical form of a biochemical model, but genotype-to-phenotype and phenotype-to-fitness relationships are found automatically by a neural network instead of being fitted with a parametric family restricted to biochemical functions. As D-LIM partly adopts the form of biochemical models, we now discuss how it recovers different aspects of interpretability found in the latter.

First, D-LIM is interpretable in the sense of explicit hypothesis testing: its predictive performance directly evaluates the validity of the model assumptions. D-LIM assumes that mutations in different genes act independently on gene-specific continuous molecular phenotypes, and that epistasis arises solely from a nonlinear mapping from these phenotypes to fitness. If D-LIM accurately predicts fitness, this indicates that the biological system is well described by a low-dimensional mechanistic model, whose variables correspond to the inferred effective phenotypes. Conversely, systematic prediction failures indicate the presence of additional interactions that cannot be captured within this low-dimensional framework. Specifically, using data from mutants in a metabolic pathway, we showed that D-LIM predicts epistasis between genes with the accuracy of mechanistic and pure ML models, thus validating the assumption that mutations in each metabolic gene mostly affect the properties of the enzyme encoded by this gene, and not other enzymes. This contrasts with biochemical models, which may validate additional hypotheses (e.g., the functional form of enzymatic responses), and with ML models, which would predict fitness without proving or disproving any property of the metabolic pathway. In contrast, D-LIM did not provide useful interpretation for mutation interactions in the case of physical interactions between proteins, as only the linear components could be extracted by the phenotype-to-fitness relationship. This shows that such interactions cannot be modelled by a low-dimensional model where axes are associated univocally with gene properties. Instead, other interpretable ML models, such as those using coupling coefficients between residues, must be used [34].

Second, D-LIM performs an inference of non-observed parameters. It infers effective phenotypic values, formally identified as the axis coordinates of the latent space between mutations and fitness. Although the inferred phenotypes are not *a priori* related to biological phenotypes, we demonstrated that the former could be interpreted as proxies of the latter up to a non-linear relationship. In particular, when the true phenotype-to-fitness relationship is monotonous, the model recovers the ordering between the inferred phenotypic values and the actual biological ones. However, when the phenotype–fitness relationship is not monotonic, we use spectral initialization to resolve ambiguities in the genotype–phenotype mapping. For example, in a scenario where the true landscape contains a maximum, two mutations with similar fitness in one genetic background might actually lie on opposite sides of that maximum. Using only that background, the model could incorrectly force the landscape to be monotonic. However, this would result in discontinuities in backgrounds where their effects diverge. We showed that spectral initialization prevents this mismatch by using cross-background variation to reveal non-monotonic features. This strategy does not require other data than genotype-to-fitness ones, but it is efficient only when mutation effects are measured in sufficiently diverse genetic backgrounds.

Third, as D-LIM can detect extrema in the phenotype-to-fitness relationship, it thus indicates the presence of phenotypic trade-offs. Trade-offs are common in biological systems, and often difficult to capture by purely statistical approaches. Indeed, approaches such as the SVD proposed in [17] are not capable of capturing trade-offs in the typical Fisher geometric model, whereas D-LIM, to our knowledge, is the only method tested that can do this. Other ML methods use latent spaces, where a mutation may span multiple dimensions. In LANTERN, the proximity between mutations in the latent space indicates a similarity between their effects. However, the coordinates of mutations cannot be directly interpreted as a biological property. In MAVE-NN, there is a single latent interpretable phenotype, but the approach is not suited for several phenotypes. MoCHI addresses the case of several phenotypes but does not deconvolve the effect of separate genes. In GenNet, nodes of the NN are connected according to the underlying gene network. Although this could improve interpretability, it requires extensive *a priori* biological knowledge, and the relationship with the phenotypes remains elusive.

Finally, if provided with some phenotype values, D-LIM can perform extrapolation beyond the initial training domain, which is usually possible with biochemical models but not with statistical ones. Extrapolation was performed in the latent space assuming continuity in the phenotype, by fitting the relationship between the inferred versus actual phenotypes with a polynomial. Measuring the phenotype of new mutants allows one to project them into the latent space, and then compute their fitness using the NN. This may be of practical interest, as it requires measuring phenotypes for single mutations instead of combinations thereof.

Overall, our results show that introducing constraints on the form of the model enhances the interpretability of neural networks while retaining most of their predictive power. Extensions of this approach may pursue two main lines, toward larger systems and down to molecular parameters. The first one is to address genome-wide combinatorial genetic perturbation screens. D-LIM cannot be applied as there is no obvious way to attribute ownership of perturbations to two or a few dimensions. Still, the notion of independent effective phenotypes may apply, but it would require to first automatically map mutations to these phenotypes while preserving a consistent phenotype-to-fitness map. Solving similar combinatorial problems with end-to-end differentiable neural networks is currently under investigation but is a non-obvious combinatorial task [35]. Such an extension may offer an alternative approach to genome-wide association studies (GWAS) [36]. Indeed, GWAS aim to map genetic perturbations to phenotypes, generally using independent linear models. Assuming additive mutation effects on intermediate molecular phenotypes that in turn interact non-linearly may allow one to better interpret epistatic interactions at the level of physiological phenotypes. The second one is aimed at learning interpretable molecular parameters, which the current model cannot do as it is limited to a single effective phenotype. It was shown by Otwinowski [21] that molecular binding can be inferred by decomposing the process in stability and affinity terms, in turn learned statistically. Such an approach is likely generalizable to other processes, up to the hypothesis that mutations independently affect those. At this stage, the hypotheses need to be determined by the user. However, it may become possible to automatically generate the mutations-to-phenotype mapping, ultimately allowing us to devise interpretable ML for large biological systems.

## Methods

### Spectral initialization

We now describe the application of the spectral initialization to a two-gene system, where fitness is available across pairs of mutations. To compute similarities between mutations, we used the fitness values where pairs of mutations $g_{i},g_{j}$ are measured in matching backgrounds $g_{k}\in N$, allowing us to compute Pearson correlation $\rho (g_{i},g_{j})=\rho ((F(g_{i},g_{k})|g_{k}\in N),(F(g_{j},g_{k})|g_{k}\in N))$. The latter allows us to construct one fully connected graph of mutations where the edges are weighted by the correlation coefficient $A_{ij}=\rho (g_{i},g_{j})$. We set a limit of at least 10 the number of matching genetic backgrounds so that the Pearson correlation is more robust. Then, we computed the normalized Laplacian $L_{n}=I-D^{-1/2}AD^{-1/2}$, where *I* is the identity and *D* is the degree matrix. From *L*_*n*_, we computed the eigenvector corresponding to the smallest non-zero eigenvalue $\varphi_{FV}$, called Fiedler vector. This Fiedler vector encapsulates information about the connectivity of the underlying graph [37]. We initialized the genotype-to-phenotype map φ with the values in $\varphi_{FV}$, from which we trained D-LIM at predicting the fitness. This procedure is sensitive to the similarity measure chosen because they capture different relationships. Therefore, we used the second eigenvalue $\lambda_{2}$, referred to as the spectral gap, to filter the cases where the spectral initialization is inadequate. For example, when all similarities are *A*_*ij*_ = 1, we have $\lambda_{2}=1$; whereas when all similarities *A*_*ij*_ = 0, we have $\lambda_{2}=0$. We therefore defined the following range $\lambda_{2}<0.95$ and $\lambda_{2}>0.05$ to spectral initialization, otherwise we use the random Xavier-Glorot method [29].

However, because of the nature of Pearson correlation, some landscapes are typically not well processed. For example, the Pearson correlation between two mutations on an additive landscape is always 1, therefore, the similarity matrix is full of ones, which does not inform us about relationships between mutations. In contrast, similarity measured with cosine similarity or Gaussian kernel distance can distinguish mutations in the additive landscape. However, our tests showed that Pearson correlation performs in general better than the other two:

- Cosine similarity is $\text{sim}(g_{i},g_{j})=\frac{g_{i}\cdot g_{j}}{\left\|g_{i}\|\|g_{j}\right\|}+1$, where ***g***_***i***_ and ***g***_***j***_ are vectors representing fitness values across matching background environments.
- Similarity based on Gaussian kernel distance is $E(g_{i},g_{j})=\text{exp}(-∥g_{i}-g_{j}∥)$.

However, there are cases of symmetric landscapes as shown in Fig J in S1 File, where no similarity measure can differentiate two mutations.

We now discuss the robustness of the spectral initialization. This initialization is sensitive to how well the fitness data covers mutations and the noise in fitness measurements. To demonstrate the first point, we used the artificial dataset generated with the tilted Gaussian model, where we trained D-LIM with increasing amounts of data points. We found that only 35% of pairwise measurements enabled us to achieve an accuracy of ρ=0.84. Although the result depends on the underlying fitness landscape, it indicates that complete measurements are not required to obtain a useful starting point. Next, we evaluated the sensitivity of the initialization to noise in the fitness measurements. To do so, we incrementally added noise to the similarity matrix using the same artificial dataset. We found that the benefit of the spectral initialization vanished when noise reached ~5 times the variance (see Fig K in S1 File).

### Models comparison

- **Additive latent model (ALM)**: This model used an unconstrained additive latent space, similar to the one proposed in LANTERN. However, here, we used a neural network to predict fitness out of the latent space. Here, we used an 2-dimension latent space, 2 layers of a 32-unit neural network for fitness prediction. We trained the model for 300 steps, using ADAM and a learning rate of 0.01 and a regularization strength of 0.001. The batch size used here is 64.
- **LANTERN**: This model used an additive latent space, where the importance of each dimension is controlled explicitly. The fitness is predicted using the Gaussian process. Here, we used *K* = 4 latent dimension and a neighborhood number of 200, optimized for 10^3^ steps using ADAM and a learning rate of 0.001.
- **MAVE-NN**: This model introduced a biophysical interpretation using a latent phenotype and a deterministic genotype to phenotype map, combined with a stochastic measurement process. Here, we used an additive genotype-to-phenotype map along with the skewed-t noise model for the measurement process. The model has been trained for 10^3^ steps with a learning rate of 0.001 and a batch size of 64.
- **Linear regression (LR)**: The linear regression is an additive map $F_{ij}=\sum_{i\in Mut_{1}}\sum_{j\in Mut_{2}}(\varphi_{i}+\varphi_{j})$. Each mutation is represented by a learnable variable. We optimized the model using Adam for 300 steps with a learning rate of 0.01 and a batch size of 64.

### D-LIM hyperparameter and loss function

To train D-LIM, we use the Gaussian negative log likelihood:


$$ℒ=\frac{1}{2}\left[\text{log}(\sigma )+\frac{(\hat{F}-F{)}^{2}}{\sigma }\right]+\lambda |\varphi |,$$ (1)

where $\hat{F}$ and σ are D-LIM predictions respectively for the fitness and its uncertainty, while λ regularizes the phenotype values.

The summary of hyperparameters for all datasets is listed as follows:

**Table:** 

	Simulated dataset	Yeast dataset	Mutation-environment dataset	Protein-protein interactions dataset
Hidden layers	[32]	[32]	[64, 64]	[64, 64]
Learning rate	1e-3	1e-3	1e-3	1e-3
Weight decay	1e-4	1e-4	1e-3	1e-4
Maximum epochs	300	400	200	100
Batch size	32	32	128	256
λ	0.001	0.01	0.01	0.01
Dropout rate	0.2	0.2	0.2	0.2
Patience of early stop	10	10	10	10

We trained D-LIM on a machine equipped with 104 Intel(R) Xeon(R) Gold 6230R CPUs at frequency 2.1HZ, where the learning typically finished in 15.62 seconds for 300 learning steps in applying yeast fitness data, see comparisons in Table A in S1 file. We implemented D-LIM using the deep learning backend PyTorch [38], while the analyses were performed using the routines implemented in the package Numpy [39].

### Statistical test

To evaluate the prediction accuracy, we employ Pearson’s correlation coefficient (ρ) as a primary metric. With only ~30 datapoints in the validation set, this approach allows us to detect a correlation of ρ=0.5 with a significance level of α=0.05 [40]. This motivated our choice of training and validation split as 30% of the datasets, we considered yielded sizes well above 30 data points. However, correlation is often overoptimistic as it only measures how well the trend is recovered. Thus, we provided along with it the corresponding coefficient of determination *R*^2^ to quantify how much of the observed variability is captured.

To evaluate inference accuracy for effective phenotypes, we used Spearman’s rank correlation because D-LIM recovers phenotypes only up to an arbitrary monotonic, potentially non-linear, transformation. This transformation accounts for the negative correlation.

To compare two models, we performed random cross-validation with more than 30 independent runs per model. We then applied a t-test to evaluate whether one model achieved a higher mean accuracy (e.g., Pearson correlation ρ), assuming normally distributed Pearson correlations.

## Supporting information

S1 File Table A. CPU time for different algorithms in fitness prediction in Env1. **Fig A.** Simultaneous fitting of fitness and inference of phenotypes. **Fig B.** Phenotype inference with and without spectral graph initialization. **Fig C.** Extrapolation of a non-monotonic fitness landscape. **Fig D.** Additivity of fitness measurements. **Fig E.** Protein–protein epistasis modeling. **Fig F.** Predicting mutation effects across varying environments. **Fig G.** First eigenvector of SVD applied to fitness data. **Fig H.** Spectral initialization performance on simulated and experimental datasets. **Fig I.** Convergence of validation loss with and without spectral initialization. **Fig J.** Similarity of mutational effects across different fitness landscapes. **Fig K.** Impact of spectral initialization on prediction accuracy.
(PDF)
