# Supplementary material for: D-LIM: A neural network for interpretable gene–gene interactions
Source: PLoS Comput Biol. 2026 Mar 23;22(3):e1014107. doi: 10.1371/journal.pcbi.1014107 (PMC13029791; doi:10.1371/journal.pcbi.1014107)
Supplement: S1 File — Fig A. Simultaneous fitting of fitness and inference of phenotypes. Fig B. Phenotype inference with and without spectral graph initialization. Fig C. Extrapolation of a non-monotonic fitness landscape. Fig D. Additivity of fitness measurements. Fig E. Protein–protein epistasis modeling. Fig F. Predicting mutation effects across varying environments. Fig G. First eigenvector of SVD applied to fitness data. Fig H. Spectral initialization performance on simulated and experimental datasets. Fig I. Convergence of validation loss with and without spectral initialization. Fig J. Similarity of mutational effects across different fitness landscapes. Fig K. Impact of spectral initialization on prediction accuracy. (PDF) [file pcbi.1014107.s001.pdf]

Legend for S1 File

Table A. CPU time for different algorithms in fitness prediction in Env1.
Fig A. Simultaneous fitting of fitness and inference of phenotypes.
Fig B. Phenotype inference with and without spectral graph initialization.
Fig C. Extrapolation of a non-monotonic fitness landscape.
Fig D. Additivity of fitness measurements.
Fig E. Protein–protein epistasis modeling.
Fig F. Predicting mutation effects across varying environments.
Fig G. First eigenvector of SVD applied to fitness data.
Fig H. Spectral initialization performance on simulated and experimental datasets.
Fig I. Convergence of validation loss with and without spectral initialization.
Fig J. Similarity of mutational effects across different fitness landscapes.
Fig K. Impact of spectral initialization on prediction accuracy.

1 Synthetic datasets

We generated synthetic data using three evolutionary and biophysical model. The linear regulation model proposed in [1] is as follows:

$$F(X,Y) = \left(w + \mu\varphi - \frac{\nu}{1/\eta - \varphi}\right)(1 - \theta_X X - \theta_Y Y), \quad (1)$$

where , $\varphi = \frac{1}{1/X+1/Y+\eta}$ denotes for flux, η is the inverse of the maximal flux φ, θ_X and θ_Y represent the cost of increasing cellular enzyme activity, w describes the growth rate, μ and ν are two variables related to downstream enzyme properties.

The tilted Gaussian model [2] is a Fisher's Geometric Model for studying genetic evolution. It is formulated as follows:

$$F = \exp(-(X,Y)M\,(X,Y)^{\mathrm{t}})/(2\pi\det(M)) \quad (2)$$

where $M = \begin{bmatrix} \frac{cos\theta^2}{2} + 2sin\theta^2 & -\frac{-sin(2\theta)}{4} + sin(2\theta) \\ -\frac{-sin(2\theta)}{4} + sin(2\theta) & \frac{sin\theta^2}{2} + 2cos\theta^2 \end{bmatrix}$, θ represents the rotation angle.

The cascade model [3] is used to study the interaction between two genes: LacI and TetR, in a transcriptional signaling cascade in E. coli. For each interaction, it follows a hill function:

$$f(M,m,a,b,n) = \frac{M-m}{1+(a/b)^n} + m.$$

$$TetR(ara) = f(M_{AraC}, m_{AraC}, [ara], K_{AraC}, n_{AraC}),$$

$$LacI(TetR) = f(M_{TetR}, m_{TetR}, TetR, K_{TetR}, n_{TetR})$$

$$Out(LacI) = f(M_{LacI}, m_{LacI}, LacI, K_{LacI}, n_{LacI})$$

where M_i and m_i are the maximum and minimum expression level, n_i shows the cooperativity. K_i is the dissociation constant, K_{AraC} is default to be 1, K_{LacI}, K_{TetR} can be considered as the phenotype variables to indicated the activity of gene LacI and TetR. If we summed up, the output will be:

$$output = F(K_{LacI}, K_{TetR}, ara) = Out(LacI(TetR(ara))),$$

The sign epistasis will be:

$$F(X,Y) = F(X,Y,ara_{min}) - F(X,Y,ara_{max})$$

1.1 Extrapolation by using latent variables

We tested here the extrapolation strategy using a non-monotonous fitness function. First, we sampled from the uniform distribution: $\phi_1, \phi_2 \sim U_{[0,5]}, i \in \{1, 2 \cdots, 36\}$, representing the true phenotype values. To validate the trained models, we extracted the data points whose phenotype values lay in $\phi_1 \in [1.5, 2.7]$ and $\phi_2 \in [1.5, 2.7]$. The other data points were used for training, as shown in Fig C.

Table A: CPU time for different algorithms in fitness prediction in Env1 of [1]. Each model was run five times and the results were average (in seconds).

Models	CPU time (s)
D-LIM	15.62 ± 0.10
LR	4.04 ± 0.01
ALM	14.40 ± 1.93
LANTERN	**133.90** ± 135.91
MAVE-NN	21.99 ± 2.63

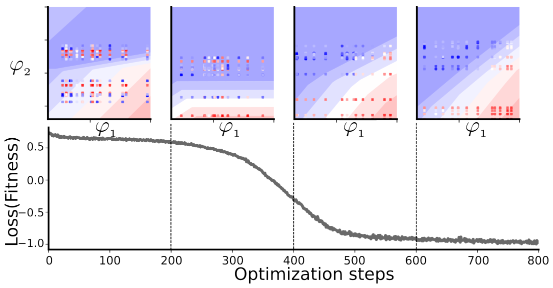

Fig. A: Simultaneous fitting of Fitness and inference of phenotypes. For the mechanistic model [1], we created artificial mutations that were randomly initialized in the inferred phenotype space of D-LIM (first of the four top sub-panels). The real fitness values of each mutant (represented by the color of the dots) did not align with the landscape predicted by D-LIM (the blue-red gradient color); because neither of the parameters of the fitness predictor, nor the inferred phenotype were yet optimized. We monitored the evolution of fitness prediction quality (Loss(Fitness)) and the inferred phenotypes across three-time points, specifically after 200 steps of ADAM optimization. During this optimization process, both the parameters of the fitness predictor and the coordinates in the latent space were adjusted and refined.

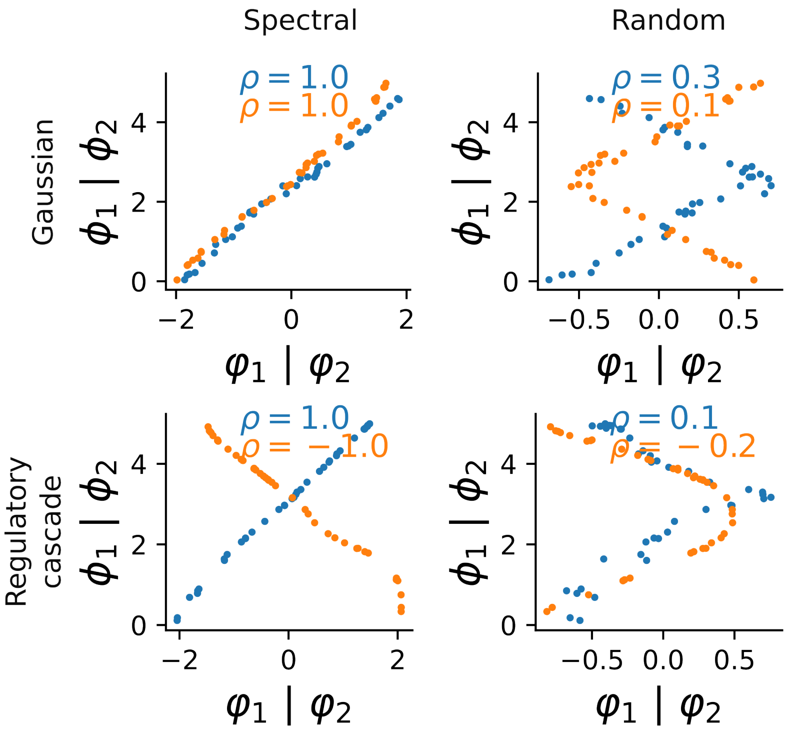

Fig. B: Phenotype inferred with (Spectral) and without spectral graph initialization (Random) for the Gaussian and the regulatory cascade models.

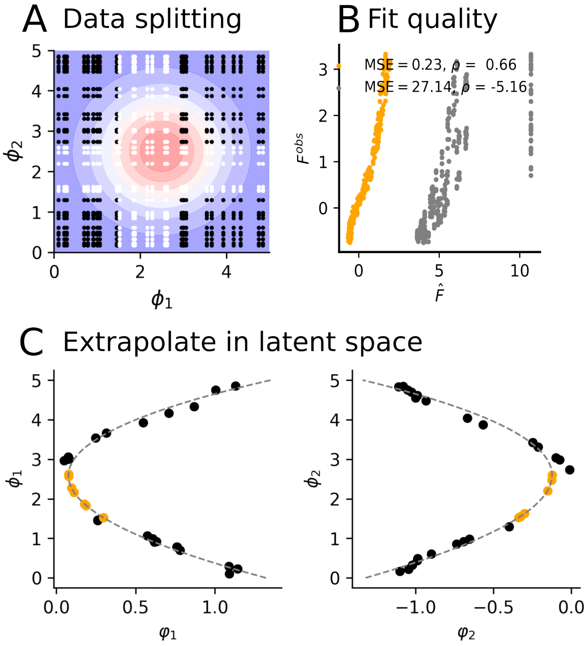

Fig. C: Extrapolation of a non-monotonic fitness landscape. A) Gaussian-type fitness landscape centered at $\phi_1 = 2$ and $\phi_2 = 2$. Black points are the training points whereas the white ones are the validation points. B) Extrapolation using new ϕ_1 (left), ϕ_2 (right) phenotypic measurements. Black points are (ϕ_1, φ_1) pairs used to parametrize the polynomial fit in a dotted grey line. Orange points are the converted ϕ_1 and ϕ_2 only seen in the validation, which are converted into latent variables using the polynomial fit. C) Fit quality. After converting the phenotypic values only seen in the validation to latent parameters: blue points are the conversion whereas grey ones are after.

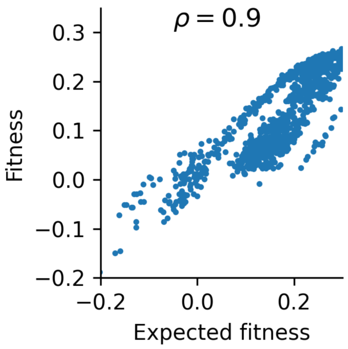

Fig. D: The fitness measured in [1] is largely additive. We compared the double mutant fitness measured $F(A, B)$ with the sum of single mutant fitness $F(A) + F(B)$.

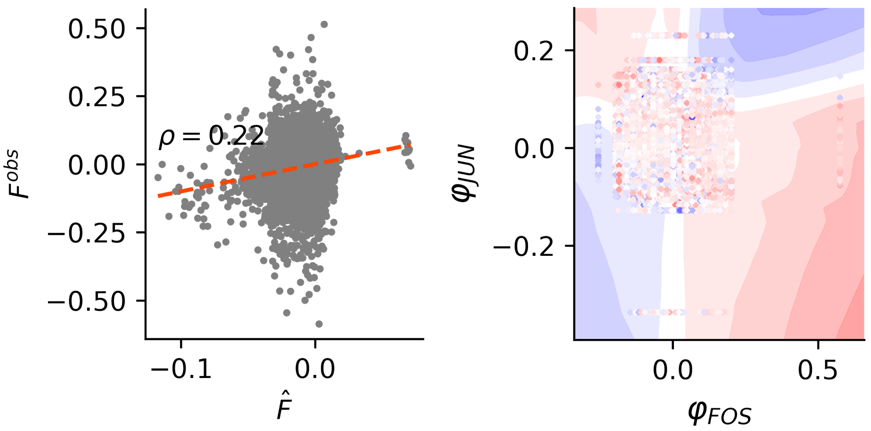

Fig. E: Protein-protein epistasis modeling. The left panel shows the performance of D-LIM for epistasis prediction. The right panel shows the inferred landscape with respect to the D-LIM phenotypes $\varphi^{JUN}, \varphi^{FOS}$.

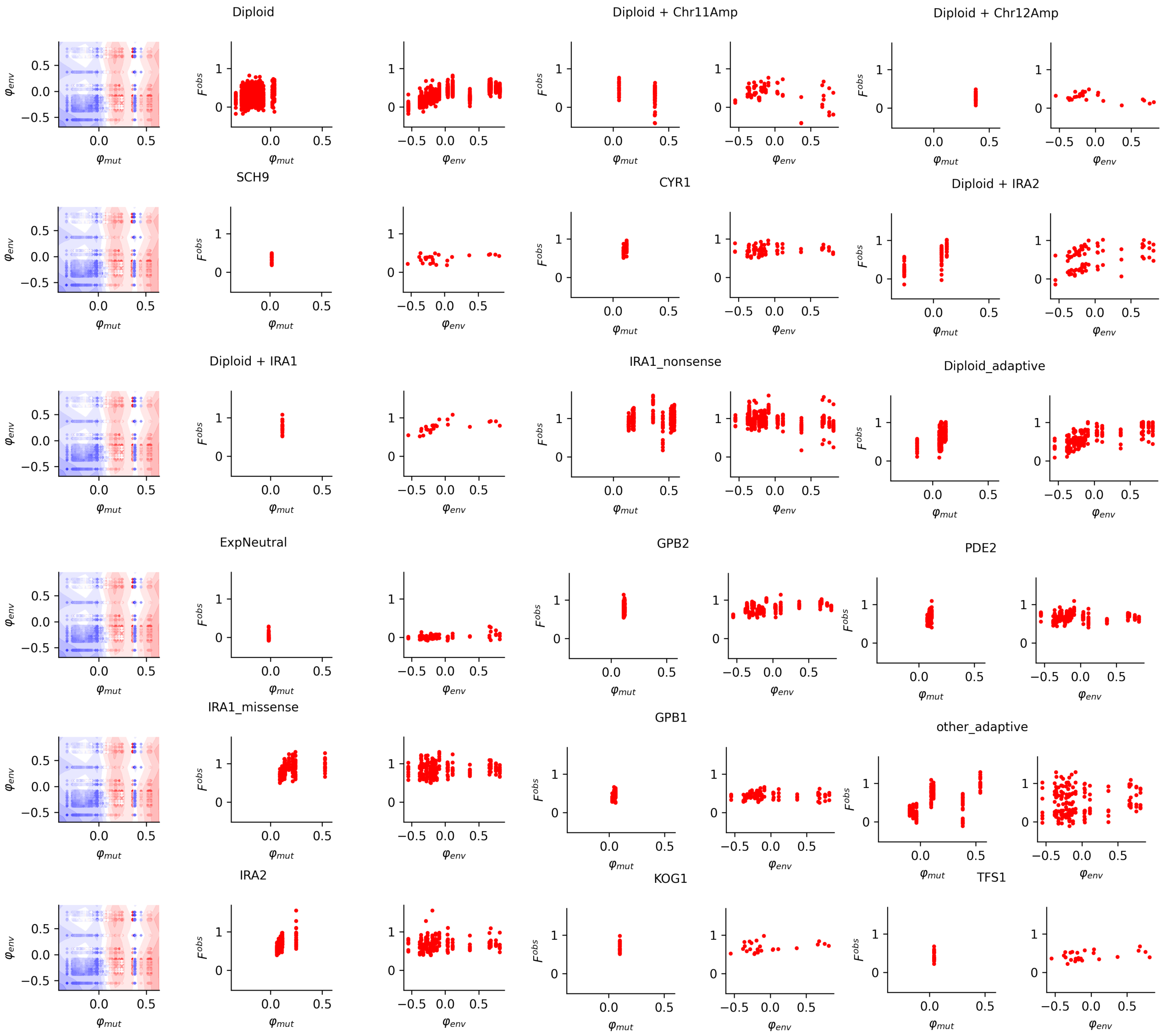

Fig. F: Predicting mutation effects across varying environments. The first column shows the inferred fitness landscape. The second, fourth, and sixth columns highlight mutations in specific genes, plotting inferred phenotypes against observed fitness to assess prediction accuracy. The third, fifth and seventh columns illustrate the environment-dependent fitness variation of mutations within the same genes across different environmental conditions.

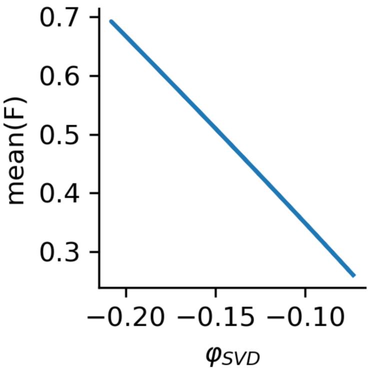

Fig. G: First eigenvector φ_{SVD} of the SVD applied to the fitness data. Using simulated data from the Fisher geometric model, we compute the φ_{SVD} and compare it to the mean fitness (mean(F)) across mutations.

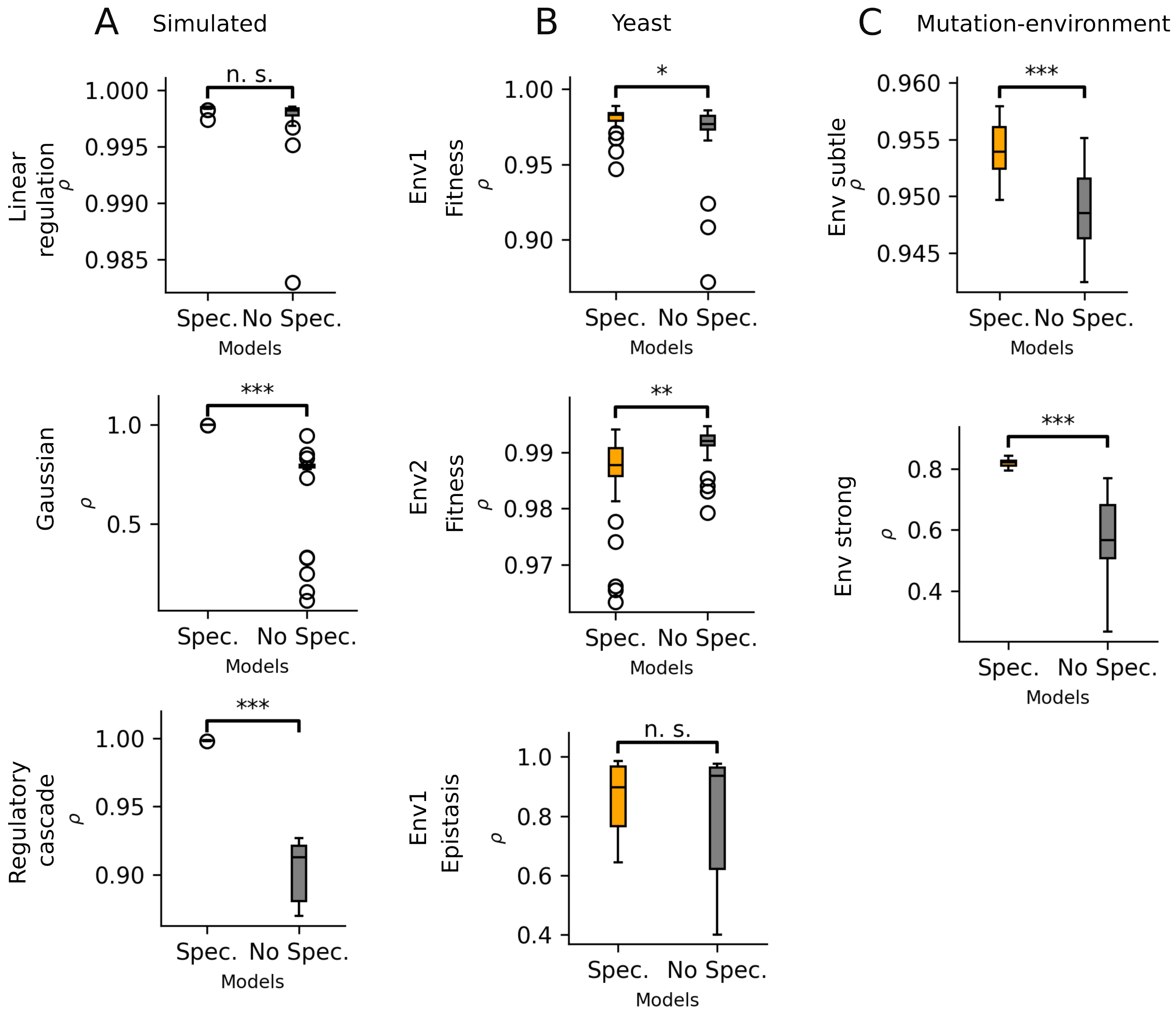

Fig. H: Comparing the spectral initialization on simulated datasets and experimental datasets (Yeast and mutation-environment datasets). It displays the Pearson correlation between predicted and observed fitness on 30 independent model trainings. A) Simulated datasets: Linear regulation, Gaussian, and regulatory cascade system. B) Yeast datasets: fitness data in Environment 1 (Fitness Env1), fitness data in Environment 2 (Fitness Env2), epistasis data in Environment 1 (Epistasis Env1). C) Mutation-environment dataset: all mutation data in subtle environments (Env subtle) and all mutation data in strong environments (Env strong).

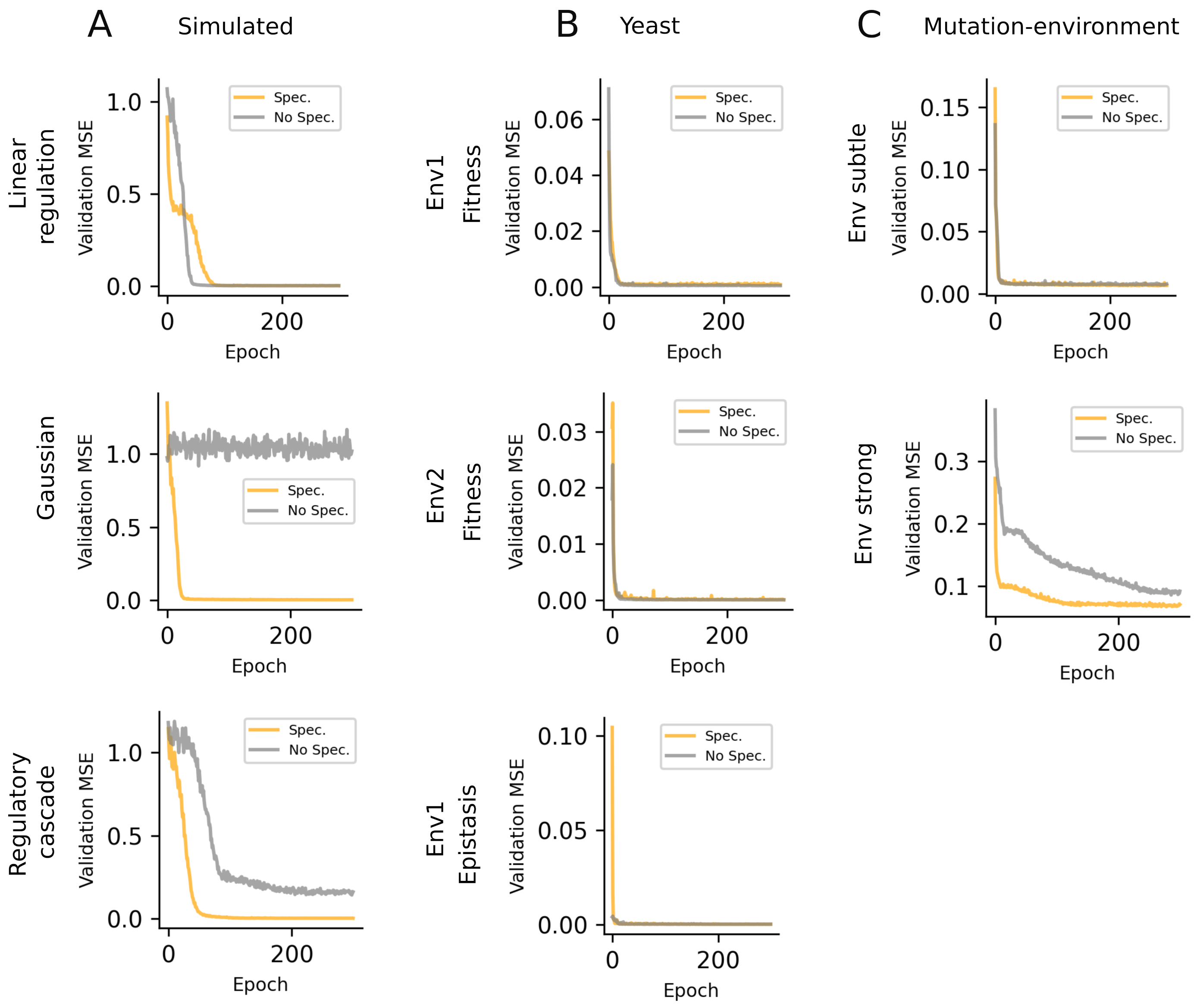

Fig. I: Comparing the convergence of validation loss by model with and without spectral initialization on simulated datasets and experimental datasets (Yeast and mutation-environment datasets). Model with and without spectral initialization start from a high loss and then converge to a low loss. A) Simulated datasets: Linear regulation, Gaussian, and regulatory cascade system. B) Yeast datasets: fitness data in Environment 1 (Fitness Env1), fitness data in Environment 2 (Fitness Env2), epistasis data in Environment 1 (Epistasis Env1). C) Mutation-environment dataset: all mutation data in subtle environments (Env subtle) and all mutation data in strong environments (Env strong).

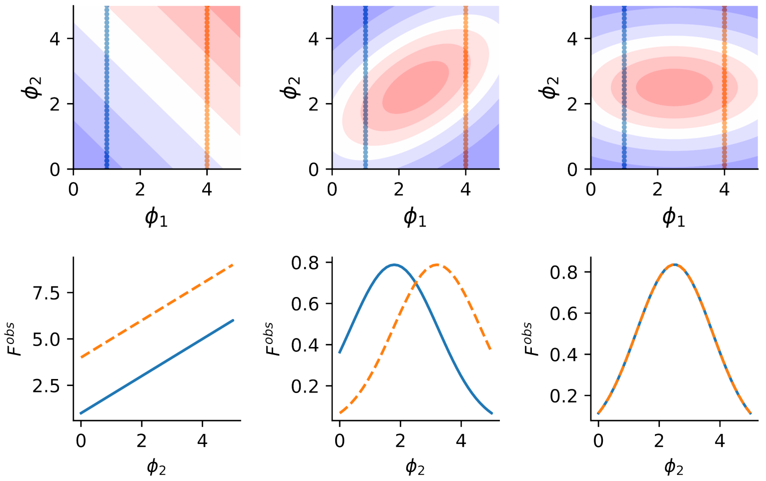

Fig. J: Similarity of mutational effects across different fitness landscapes. (Left) The top panel shows the contour of an additive model landscape, with two mutations in ϕ_1 and ϕ_2 highlighted in blue and orange. The bottom panel displays the corresponding fitness profiles of these mutations across different genetic backgrounds. (Center and right) The same configuration is shown for a rotated and a non-rotated Fisher geometric model, respectively, illustrating how mutational effects vary with landscape geometry.

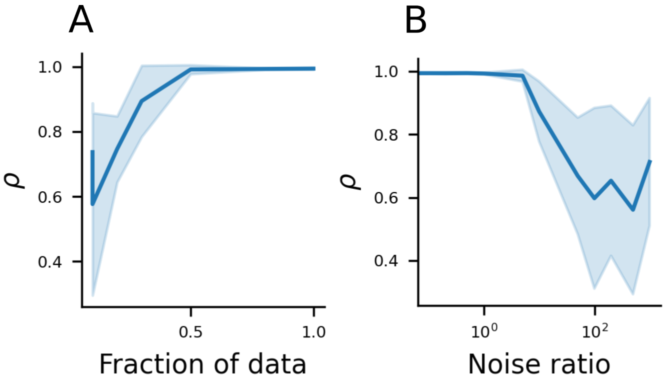

Fig. K: Impact of spectral initialization on prediction accuracy using the simulated data from the tilted Gaussian model. A) Robustness of spectral initialization to data availability. The x-axis represents the fraction of data used to construct the similarity matrix during spectral initialization, and the y-axis shows the Pearson correlation between model predictions and experimental measurements. B) Robustness of spectral initialization to noise. Gaussian noise was added to the similarity matrix during initialization. The x-axis indicates the ratio between the variance of the injected noise and the variance of the original data. The y-axis shows the resulting Pearson correlation between predicted and measured fitness values.

References

[1] Harry Kemble, Catherine Eisenhauer, Alejandro Couce, Audrey Chapron, Mélanie Magnan, Gregory Gautier, Hervé Le Nagard, Philippe Nghe, and Olivier Tenaillon. Flux, toxicity, and expression costs generate complex genetic interactions in a metabolic pathway. *Science Advances*, 6(23):eabb2236, 2020.

[2] Olivier Tenaillon. The utility of fisher's geometric model in evolutionary genetics. *Annual review of ecology, evolution, and systematics*, 45:179–201, 2014.

[3] Philippe Nghe, Manjunatha Kogenaru, and Sander J Tans. Sign epistasis caused by hierarchy within signalling cascades. *Nature communications*, 9(1):1451, 2018.
